# Design, simulation, and testing of a tunable MEMS multi-threshold inertial switch

**DOI:** 10.1038/s41378-024-00662-z

**Published:** 2024-03-07

**Authors:** Qiu Xu, Rodrigo T. Rocha, Yousef Algoos, Eric Feron, Mohammad I. Younis

**Affiliations:** 1 Faculty of Electronic Information Engineering, Huaiyin Institute of Technology, Huai’an, 223003 PR China; 2 Computer, Electrical and Mathematical Science and Engineering Division, King Abdullah University of Science and Technology, Thuwal, 23955-6900 Saudi Arabia; 3 Piezoelectric Microsystem Technologies, Silicon Austria Labs, Villach, 9524 Austria; 4 Physical Sciences and Engineering Division, King Abdullah University of Science and Technology, Thuwal, 23955 Saudi Arabia; 5 Mechanical Engineering Department, State University of New York, Binghamton, NY 13902 USA

**Keywords:** Sensors, Electrical and electronic engineering

## Abstract

This paper presents a tunable multi-threshold micro-electromechanical inertial switch with adjustable threshold capability. The demonstrated device combines the advantages of accelerometers in providing quantitative acceleration measurements and g-threshold switches in saving power when in the inactive state upon experiencing acceleration below the thresholds. The designed proof-of-concept device with two thresholds consists of a cantilever microbeam and two stationary electrodes placed at different positions in the sensing direction. The adjustable threshold capability and the effect of the shock duration on the threshold acceleration are analytically investigated using a nonlinear beam model. Results are shown for the relationships among the applied bias voltage, the duration of shock impact, and the tunable threshold. The fabricated prototypes are tested using a shock-table system. The analytical results agree with the experimental results. The designed device concept is very promising for the classification of the shock and impact loads in transportation and healthcare applications.

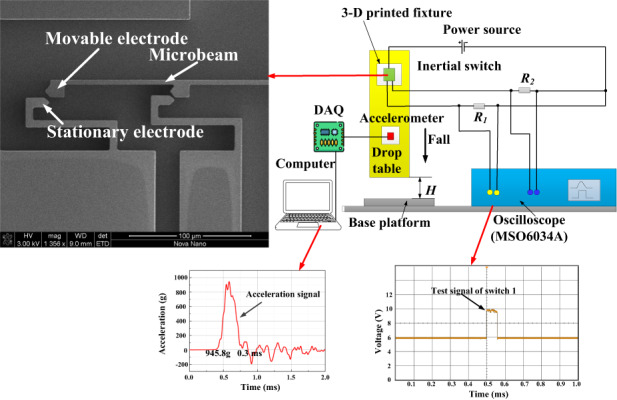

## Introduction

Inertial switches based on micro-electromechanical systems (MEMS) technology have been widely used in civilian and military fields such as healthcare, video games, transportation, safety-and-arming systems, and the automotive field. This is due to their advantages of a small size, low cost, zero power in the untriggered state, and mass production^1–4^. The working principle of an inertial switch is as follows: the proof mass (i.e., movable electrode) quickly moves along the sensing direction and contacts the stationary electrode when the device is subjected to an acceleration beyond the threshold level, forming an external electrical circuit^5^. Subsequently, the proof mass rapidly is drawn back to its original position due to the restoring force of the spring, turning off the external circuit. Therefore, the inertial switch has the great advantage of acting like an open circuit when not active in the OFF state; it consumes power only when the threshold is exceeded (near zero power consumption). With the emergence of the Internet of Things (IoT), tremendous amounts of inertial sensors are increasingly in demand to meet the market’s needs^6–8^. In most cases, inertial switches are designed to have a single threshold which can only provide binary “ON/OFF’ information^9–15^. However, they are incapable of providing quantitative information on the experienced acceleration^16–21^, which is desirable for certain applications, such as for brain impact injuries to classify the severity of injury based on the experienced shock level. Therefore, it is desirable to develop a multi-threshold inertial switch that can provide more quantitative data while retaining the attractive power-saving advantage of inertia switches^13–15,22^.

To realize different threshold levels, one method is to design a structure that makes contact with various standing switches separated at various distances from a moveable electrode. In recent work, Niyazi et al.^23^ proposed a bidirectional multi-threshold inertial switch, where stationary electrodes are placed at different displacements in two directions so that the various thresholds are generated. Xu et al.^24^ reported a high-g biaxial inertial switch with three threshold levels, the threshold varies within the range of 800–2600 g and senses the acceleration on each side of a proof mass (+*x*, −*x*, +*y*, and −*y* directions).

Active tuning of the threshold acceleration can also be used to achieve various threshold accelerations^25–28^. Younis et al. first proposed the adjustable threshold capability of single-threshold inertia switches based on the softening effect of the electrostatic force in ref. ^29^ and subsequently experimentally demonstrated this capability in refs. ^30,31^. Kim et al. proposed an inertial switch with a bi-directional adjustable threshold function. Its threshold value increases from 2.0 to 17.25 g when a bias voltage of 30 V is applied to the pushing comb and pulling comb, respectively^32,33^.

Among the recent contributions in inertial switches, Ren et al.^2^ designed a self-powered inertial switch with a single threshold, which could be triggered at the threshold acceleration of 40 g, making it binary with ON-OFF information. Reddy et al.^34^ developed a zero-power shock sensor with multiple threshold accelerations. The latching part of the proof mass latches the various discrete latch positions based on the applied external impact, which can measure quantitatively the acceleration in the interval of 20–250 g by inspecting the latching position under a microscope. However, this kind of readout is not practical and limits its application. Zhang et al.^35^ reported a bi-directional tunable inertial switch, which can detect acceleration in a wide range from 79 to 13 g when a bias voltage applied to the bottom plate increases from 0 to 100 V. Kumar et al.^36^ demonstrated a tunable acceleration threshold switch connected to a microcontroller, which applies a search algorithm onto a set of electrostatic actuators to detect the range of magnitudes of the applied acceleration. This design consumes less power than MEMS accelerometers. However, the microcontroller still consumes power continuously to apply the utilized algorithm.

As can be noted, and summarized in Table 1, the previous literature focuses on single thresholds or fixed thresholds that are not tunable. In addition, some device concepts do not allow expanding the number of thresholds as much as needed. Some works have complex transduction and readout methods. Therefore, in this work, we propose a tunable multi-threshold inertial switch based on a cantilever-type microbeam, aiming to classify the magnitude of acceleration while saving power in the untriggered state. This design can be expanded to accommodate more thresholds as needed, so the same device principle can be extended for 3 bits, 4 bits, or a higher number of bits. As highlighted in Table 1, the proposed design can overcome previous designs’ disadvantages. The tunability of the switch and the effect of the shock duration on the threshold are discussed. The microcantilever beam is modeled as an Euler-Bernoulli beam and its response is investigated through numerical simulations. Then, fabricated prototypes are experimentally characterized and tested using stroboscopic video microscopy and a shock-table system.

**Table 1:** A comparison of the performance of different inertial switches

Device	Ren et al.^2^	Reddy et al.^34^	Zhang et al.^35^	Kumar et al.^36^	This work
Type	Binary	Multiple thresholds	Tunable	Tunable	Multiple thresholds and tunable
Tunability	No	No	Yes	Yes	Yes
Multi-threshold	No	Yes	No	No	Yes
Number of bits	1	10	1	1	2
Design expandable to accommodate more thresholds	No	Yes	No	Yes	Yes
Output	Digital	No transduction	Digital	Generated by microcontroller	Separate, digital
Read out	Physical electrical switch (Simple)	Optical (Complex)	Physical electrical switch (Simple)	Needs electrostatic actuators (Complex)	Physical electrical switch (Simple)
Detect acceleration arrange	/	20–250 g	13–79 g	0–1 g	1085–1600 g

The rest of the paper is organized as follows. “Device design and working principle” provides the new device concept design and demonstrates the working principle. “Mathematical model” presents the mathematical model of the microcantilever beam. “Characterization and testing” describes the experimental setup and characterization of the microbeam. In “Numerical simulations”, the dynamic response of the electrostatically actuated device under different shock levels is theoretically investigated through numerical simulations. “Acceleration threshold test” presents the experimental data and compares them with the simulated results. “Conclusion” presents concluding remarks.

## Device design and working principle

**Fig. 1:**
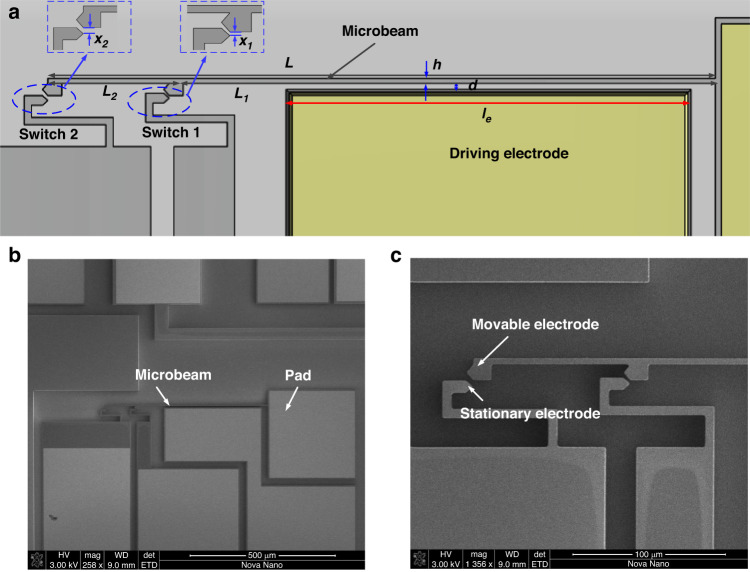
Schematic of the fabricated device. **a** Sketch of the tunable multi-threshold inertial switch. **b** SEM image of the fabricated microbeam. **c** Close-up image of the movable and stationary electrodes

The main components of the device are a micro cantilever beam, a driving electrode, switch 1, and switch 2, Fig. 1a. The device was fabricated by MEMSCAP through the SOIMUMPs process. The sensitive direction of the device is perpendicular to the substrate. The microbeam has length *L* = *L*_*1*_ + *L*_*2*_, depth *b*, and thickness *h*. The distances between the movable and stationary electrodes of switches 1 and 2 are *x*_*1*_ and *x*_*2*_, respectively. When a switch is subjected to an acceleration at or above the designed threshold levels, the movable electrode quickly moves forward and touches the corresponding stationary electrode. The part length (*L*_*1*_) of the beam forms one side of the parallel-plate electrodes, which is separated from another anchor with a gap distance of *d*. When the bias voltage is applied on two parallel electrodes, the electrostatic actuation is generated and the adjustment of the threshold value is realized. The main nominal dimensions for the designed switch are listed in Table 2. Table 3 shows the material properties of the proposed device.

**Table 2:** Main geometric parameters of the designed cantilever microbeam

Components	Geometric parameters	Value (µm)
Beam	Length *L*_*1*_	436
	Length *L*_*2*_	114
	Thickness *h*	3.3
	Depth *b*	25
Switches 1 and 2	Gap *x*_*1*_	2
	Gap *x*_*2*_	4
Driving electrode	Length *l*_*E*_	356
	Air gap *d*	4.8
Mass dimensions	*a*_*1*_	10.5
	*a*_*2*_	11.5
	*a*_*3*_	6.8

**Table 3:** Material properties of silicon

Description	Parameter	Value
Young’s modulus	E	170 GPa
Density of silicon	*ρ*	2329 kg/m^3^

## Mathematical model

The microcantilever beam in Fig. 1a is modeled as a continuum system through Hamilton’s principle^37,38^, yielding the equations of motion below

1 $$\begin{array}{c}{\ddot{\hat{w}}}_{1}+2{\zeta }_{1}{\dot{\hat{w}}}_{1}+{\hat{w}}_{1}^{IV}=\beta \frac{{({V}_{DC}+{V}_{AC}\cos \Omega t)}^{2}}{{(1-{\hat{w}}_{1})}^{2}}U(\frac{{l}_{E}}{{L}_{1}}-\hat{x})-\alpha \ddot{S}\\ {\ddot{\hat{w}}}_{2}+2{\zeta }_{2}{\dot{\hat{w}}}_{2}+{\omega }_{2}^{2}{\hat{w}}_{2}^{IV}=-\alpha \ddot{S}\end{array}$$

where the boundary conditions and coefficients are expressed in Eqs. (S16) and (S17a–c) in the Supplementary Materials along with the equation derivation.

Next, considering five modes of vibration, we reduce Eq. (1) by using

2 $${\hat{w}}_{n}(\hat{s},\hat{t})=\mathop{\sum }\limits_{m=1}^{5}{\phi }_{nm}(\hat{s}){u}_{m}(\hat{t})$$

where *n* is the beam element index and *m* is the mode of vibration index, and by applying the Galerkin method. Then, the modes of vibration for each beam element are superimposed to obtain the reduced-order equations of motion, according to the principle of minimum energy. To yield the simulation results, the time-dependent reduced-order equations are integrated in time using the 4th-order Runge-Kutta numerical method with the parameters in Tables 2 and 3.

## Characterization and testing

Figure 1b, c shows SEM images of the MEMS microstructure fabricated by MEMSCAP based on the Silicon-On-Insulator Multi-User MEMS process (SOIMUMPs). A Ti/Au layer is sputtered on these fabricated devices to enhance the electrical conductivity during the test and improve the quality of the contact signal while switching.

**Fig. 2:**
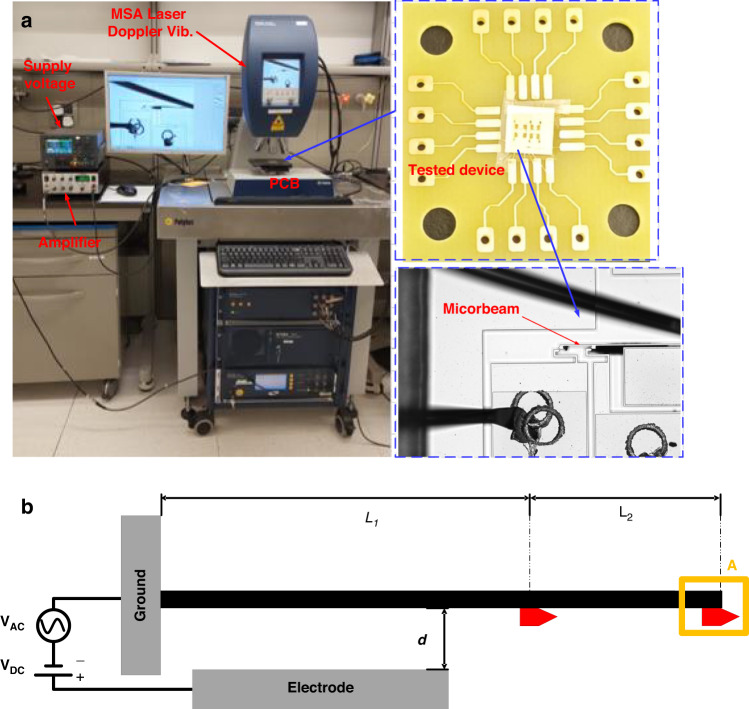
Experimental setup for the data acquisition of the microcantilever beam. **a** The MSA-500 setup with an external DC power supply and an amplifier with the device chip positioned on a stage for stroboscopic video microscopy measurements. **b** Schematic of the electrostatically actuated microcantilever beam

The in-plane resonance frequencies are measured through the stroboscopic video microscopy of the Micro System Analyzer (MSA-500) from Polytec (Fig. 2a) using a ring-down measurement through an electrostatic actuation with no shock applied. We set the camera’s target A at the tip of the cantilever, as shown in the schematic of the electrostatically actuated cantilever beam in Fig. 2b, to obtain the transversal displacements of the microbeam when subjected to an electrostatic actuation.

The frequency response curves, and forced vibration responses, are obtained via stroboscopic video microscopy with a Bode plot of the Planar Motion Analyzer (PMA) software of the MSA.

Both measurement methods are performed under environmental pressure to determine the quality factor (damping ratio) of the structure for the shock vibration analysis, which is calculated via Eq. (S20) in the Supplementary Material, obtained as *Q* = 15.58.

Next, extensive analyses are carried out by applying shock excitation along with the obtained quality factor. The shock pulse in Eq. (1) is given by

3 $$\ddot{S}=a(t)={a}_{0}\,\sin \left(\frac{\pi }{{t}_{0}}t\right)$$

where *a*_*0*_ is the acceleration amplitude, *t*_*0*_ is the shock duration, and *a*(*t*) is the acceleration for further notation.

## Numerical simulations

**Fig. 3:**
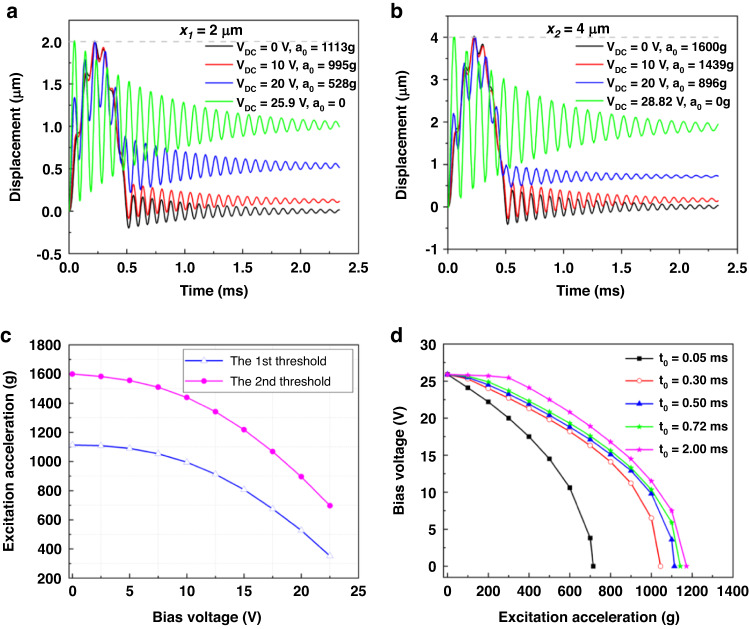
Numerical simulations of the microbeam under different shock accelerations and DC loads. Simulated time history of the displacement, in switches (**a**) 1 and (**b**) 2, under various combined values of the bias DC voltages and accelerations with duration *t*_*0*_ = 0.5 ms. **c** Numerically simulated relationship between the bias voltage and the first and second threshold accelerations. **d** Simulated results showing the bias voltage against the excitation acceleration threshold of a half-sine pulse of duration *t*_*0*_ = 0.05 ms, *t*_*0*_ = 0.3 ms, *t*_*0*_ = 0.5 ms, *t*_*0*_ = 0.72 ms, and *t*_*0*_ = 2 ms. It can be seen from the black line that the inertial switch is activated at lower thresholds in the case of *t*_*0*_ = 0.05 ms and *t*_*0*_ = 0.3 ms than in the case of *t*_*0*_ = 0.5 ms, *t*_*0*_ = 0.72 ms, and *t*_*0*_ = 2 ms. This is attributed to the fact that the microcantilever beam experiences the shock force as a dynamic load when the shock durations are *t*_*0*_ = 0.05 ms and *t*_*0*_ = 0.3 ms, while as a quasi-static load when *t*_*0*_ = 0.5 ms, *t*_*0*_ = 0.72 ms, and *t*_*0*_ = 2 ms

Figure 3a, b shows the simulated dynamic response of the cantilever beam under the combination of various DC voltages and accelerations with a duration of *t*_*0*_ = 0.5 ms. Switch 1 is just triggered by the shock impact at 1113 g, which represents the first threshold acceleration at zero DC voltage, Fig. 3a. The displacement in the steady state is approximately zero because the natural period of the microbeam (*t*_*n*_ = 0.079 ms) is much shorter than the period of the applied acceleration (*t*_*0*_ = 0.5 ms). The black line in Fig. 3a shows that the microbeam experiences a shock force as a quasi-static force. Therefore, the shape of the microbeam response is similar to the acceleration pulse profile (quasi-static response). The first threshold acceleration in switch 1 is slightly reduced to 995 g when the bias voltage V_DC_ is increased to 10 V, as shown by the red line in Fig. 3a. When the bias voltage is increased to 20 V, the first threshold acceleration in switch 1 considerably decreases to 528 g (blue line). It can be seen from the blue line that the oscillation magnitude during the residual response is close to 0.5 and larger than that for the black line at zero voltage and the red line (V_DC_ = 10 V). Because the electrostatic force in this case is greater than that at 20 V. The green line in Fig. 3a shows a response time history when actuated by V_DC_ = 25.9 V without any acceleration. The steady-state amplitude increases more. It resulted from the larger electrostatic load compared with those of 0, 10 V, and 20 V cases. The time response of switch 2 is similar to that of switch 1 (Fig. 3b).

In Fig. 3c, we show a plot of the first and second half-sine acceleration thresholds assuming a duration of 0.5 ms against the bias voltage. The first and second threshold accelerations show a considerable decrease from 1113 to 352 g and from 1600 to 697 g, respectively, when the bias voltage is increased from 0 to 22.5 V.

Next, we investigate the effect of the shock duration on the threshold acceleration under different bias voltages. Figure 3d shows a plot of the bias voltage versus the half-sine acceleration for five durations: 0.05 ms (black line), 0.3 ms (red line), 0.5 ms (blue line), 0.72 ms (green line), and 2 ms (pink line). As can be seen from Fig. 3d, the cantilever microbeam can reach the threshold acceleration more easily at the same bias voltage for *t*_*0*_ = 0.05 ms and *t*_*0*_ = 0.3 ms than for the other durations. As shown in Fig. S5 of the Supplementary Material, when the shock duration and the natural period time of the microbeam of the cantilever microbeam *t*_*0*_*/t*_*n*_ is less than 5 (in the regime from A to B), it corresponds to the dynamical loading regime in the shock spectrum. For the cases of *t*_*0*_ = 0.05 ms and *t*_*0*_ = 0.3 ms, *t*_*0*_*/t*_*n*_ are 0.63 and 3.79. Hence, both of these cases belong to the dynamic regime. However, the microbeam experiences the shock force as a quasi-static regime when the shock durations are 0.5, 0.72, and 2 ms because the ratio *t*_*0*_*/t*_*n*_ is greater than 5. These results comply with the previous results in the literature, as illustrated in Fig. S4 of the Supplementary Material.

## Acceleration threshold test

**Fig. 4:**
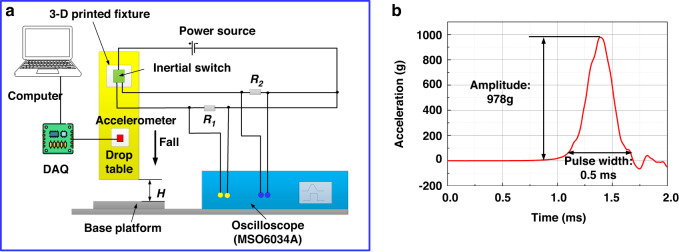
Shock-table experimental setup schematic. **a** Picture of the experimental setup of the shock-table system for testing the fabricated microbeams. **b** Example of the generated acceleration signal with an amplitude of 978 g and a duration of 0.5 ms

The fabricated prototypes are tested using a shock-table system by Lansmont, where an ADXL-193 standard accelerometer with a sensitivity of 8 mV/g is utilized to calibrate the acceleration that the device experiences, Fig. 4a. The test circuit shows the connection among a DC power supply (8 V), two divided resistances (R_1_ and R_2_) of 300 Ω, the test microbeam, and a multi-channel oscilloscope (Agilent 6000 MSO6034A). A multi-channel oscilloscope is used to simultaneously capture various signals when a switch is activated. Two colored signals are acquired from the oscilloscope: the yellow and green signals represent the contact signals of switches 1 and 2, respectively. DAQ is a data acquisition system that can capture the acceleration signal of a standard accelerometer. The switch is fixed on the lateral surface of a 3-D printed fixture to guarantee that the sensitive direction of shock impact is perpendicular to the ground. A half-sine-shaped acceleration signal with various amplitudes and durations can be generated by adjusting the starting height of the shock table and the stiffness of the shock table. When the shock table freely falls from the starting height of 15 cm, the generated acceleration is 978 g with a duration of 0.5 ms, as shown in Fig. 4b.

**Fig. 5:**
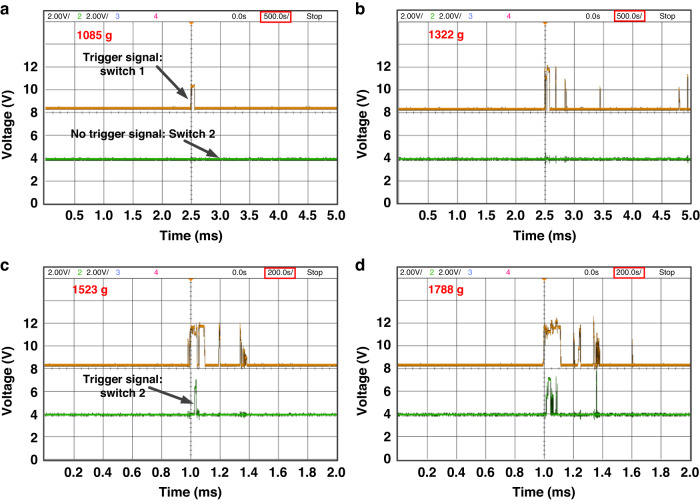
Test results of the microbeam under different shock accelerations. When the applied accelerations are (**a**) 1085 g, (**b**) 1322 g, (**c**) 1523 g, and (**d**) 1788g, respectively, at zero voltage

Figure 5 shows the measured results when the test switches are subjected to acceleration pulses with various amplitudes in the sensitive direction. The starting height is gradually increased until the yellow signal starts to appear at an acceleration pulse at 1085 g, which is found as the first threshold acceleration, Fig. 5a. A bouncing behavior is observed, as shown by the yellow signal when the acceleration increases to 1322 g, Fig. 5b. Switch 2 is just ON at 1523 g, as shown by the green signal in Fig. 5c, which indicates that the second threshold acceleration is reached. Figure 5d shows that the contact time is extended at 1788 g.

### Tunability of switch 1

**Fig. 6:**
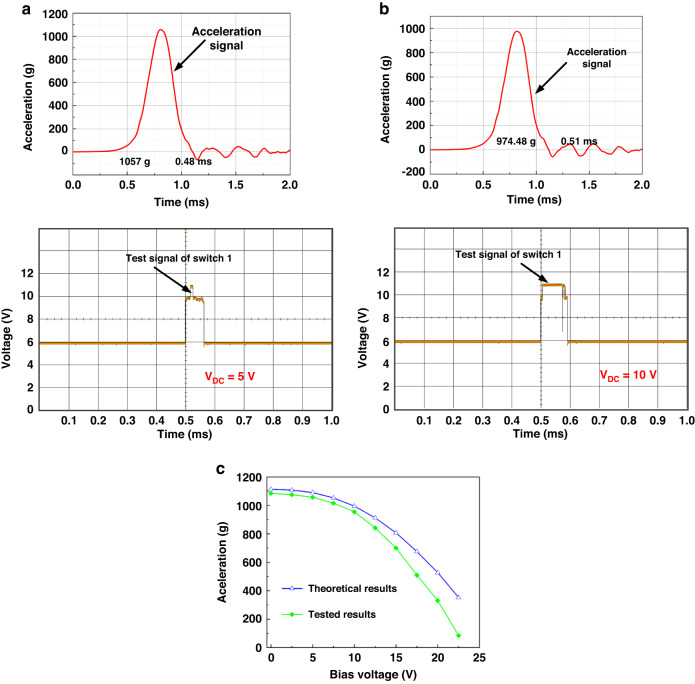
Test threshold level of the fabricated inertial switch under different shock accelerations and DC loads. When the combined loads are (**a**) a = 1057 g and V_DC_ = 5 V, (**b**) a = 974.48 g and V_DC_ = 10 V, respectively. **c** Simulated (blue) and measured (green) threshold acceleration versus the bias voltage

To investigate the effect of the bias voltage on the first threshold acceleration, Fig. 6a, b shows examples of test results under the combination of the electrostatic and acceleration forces. As seen from the yellow signal in Fig. 6a, switch 1 is just activated when the bias voltage increases to 5 V at an acceleration of 1057 g indicating reaching the first threshold acceleration in switch 1 at 5 V. Similarly, the first threshold acceleration is decreased to 974.48 g at 10 V, Fig. 6b.

To study the adjustable threshold capability, the threshold levels of switch 1 under different bias voltages are tested. Figure 6c shows the threshold acceleration as a function of the bias voltage, where the blue curve is obtained through the numerical simulations, and the green curve refers to the experimental results. It is worth noting that the experimental and simulated results show excellent agreement and exhibit the same trend: the threshold acceleration decreases with the increase of the bias voltage. The green curve shows that the test threshold acceleration is 1085 g at zero bias voltage, and the test threshold value decreases from 1085 g to 85 g when the bias voltage varies within the range of 0–22.5 V. The theoretical threshold level decreases from 1113 to 352 g in the same bias voltage regime. It is clear that the test threshold acceleration is lower than the theoretical value. Potential reasons for the deviation include fabrication imperfections (over-etching during the DRIE process; consequently, decreasing the thickness of the microbeam, thus decreasing the stiffness of the switch).

### Effect of the shock duration on the threshold acceleration

**Fig. 7:**
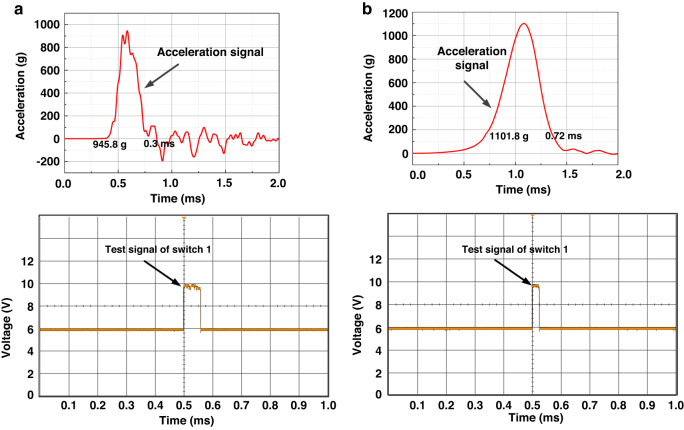
Test results of the first threshold-level acceleration of the fabricated device. When the applied acceleration is a shock pulse of (**a**) amplitude 945.8 g in the dynamic loading case of *t*_*0*_ = 0.3 ms, (**b**) amplitude 1101.8 g in the quasistatic case of *t*_*0*_ = 0.72 ms

Figure 3d shows that the numerical simulation results reveal that the threshold acceleration increases with increasing the period of shock impact. Next, we verify this conclusion experimentally. Figure 7a, b shows the first test of the threshold-level acceleration of the fabricated device for different shock pulses. As shown in Fig. 7a, the switch is turned on at a threshold acceleration of 945.8 g with a period of 0.30 ms. However, when the period of the half-sine shock increases to 0.72 ms, the test threshold acceleration increases to 1101.8 g illustrated in Fig. 7b. This indicates that a higher threshold acceleration is required to activate the switch in the case of a shock period of *t*_0_ = 0.72 ms (quasi-static range) compared to the shock period of t_0_ = 0.3 ms. It is attributed to the fact that *t*_0_ = 0.72 ms belongs to the quasi-static regime, while *t*_0_ = 0.3 ms lies in the dynamic regime as discussed in “Numerical simulations” and Fig. S5 of the Supplementary Material. Note that these results are in agreement with the numerical simulations previously presented in Fig. 3d.

## Conclusion

A multi-threshold MEMS tunable inertial switch was successfully designed, simulated, and tested. The designed device aims to provide more quantitative information on the level of applied acceleration while retaining the significant energy-saving advantages of a binary inertial switch. The dynamic response of the switch, the effect of the bias voltage, and the shock duration on the threshold acceleration were determined through theoretical analysis. Experimentally, the fabricated switch prototypes were tested using a drop-table system. The experimental results demonstrate that a multi-threshold inertial switch can provide quantitative acceleration measurements and detect accelerations ranging from 1085 to 1600 g at zero voltage. The test threshold acceleration decreases with the increase of the bias voltage, and the threshold acceleration at *t*_*0*_ = 0.3 ms is smaller than one at *t*_*0*_ = 0.72 ms. The simulation results are in good agreement with the experimental data. Future work may be directed at realizing higher resolution and at further enhancing the adjustable threshold capability of inertial switches.

### Supplementary information

Supplementary Material
