# Supplementary material for: Design, simulation, and testing of a tunable MEMS multi-threshold inertial switch
Source: Microsyst Nanoeng. 2024 Mar 7;10:31. doi: 10.1038/s41378-024-00662-z (PMC10917738; doi:10.1038/s41378-024-00662-z)
Supplement: Supplementary file 1 — Supplementary Material [file 41378_2024_662_MOESM1_ESM.docx]

**Design, Simulation, and Testing of a Tunable MEMS Multi-threshold Inertial Switch**

Supplementary Material

Qiu Xu^1,2^, Rodrigo T. Rocha^3^, Yousef.Algoos^4^, Eric Feron^2*^, and Mohammad I. Younis^4,5*^

^1^Faculty of Electronic Information Engineering, Huaiyin Institute of Technology, Huai’an, 223003, PR China

^2^Computer, Electrical and Mathematical Science and Engineering Division, King Abdullah University of Science and Technology, Thuwal, 23955-6900, Saudi Arabia

^3^Piezoelectric Microsystem Technologies, Silicon Austria Labs, Villach 9524, Austria

^4^Physical Sciences and Engineering Division, King Abdullah University of Science and Technology KAUST, Thuwal 23955, Saudi Arabia

^5^Mechanical Engineering Department, State University of New York at Binghamton 4400 Vestal Parkway East, Binghamton, NY 13902, USA

**Corresponding author:* [eric.feron@kaust.edu.sa](mailto:eric.feron@kaust.edu.sa), [myounis@binghamton.edu](mailto:myounis@binghamton.edu)

**S1. Mathematical Modeling**

The microcantilever beam of Fig. 1 is modeled as a continuum system through Hamilton’s principle [S1,S2], according to the schematic in Fig. S1. The continuum system has a reference general coordinate XOY_1_, and a second one XOY_2_ rotated in relation to the reference one.


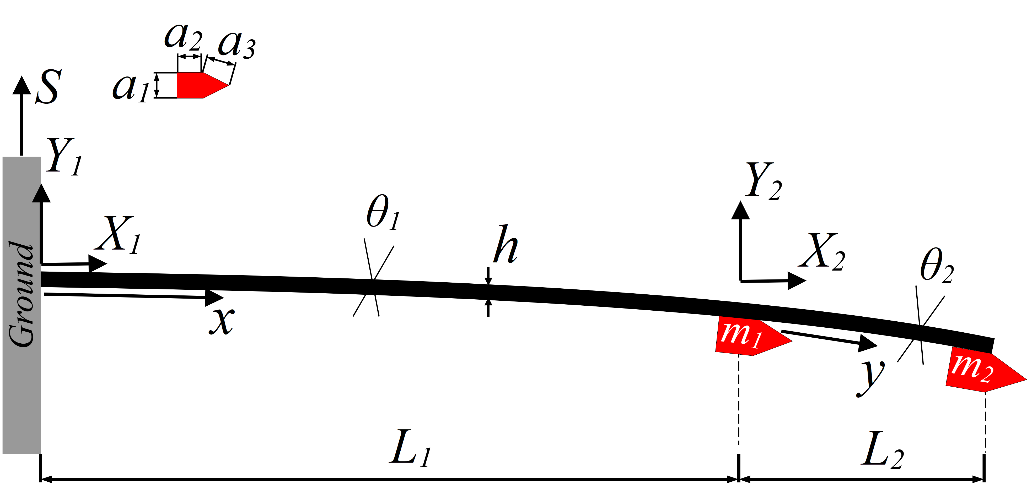


**Fig. S1** – Continuum microcantilever beam schematic.

Each coordinate system is related to the axial and transversal displacements for each beam element as:

$\begin{matrix} X_{i}=u_{i}\left(s,t\right)+s \\ Y_{i}=w_{i}\left(s,t\right) \end{matrix}$ (S1)

where *u* and *w* are respectively the axial and transversal displacements, the index “*i*” is the beam element, and *s* is the independent spatial coordinate of each beam element. Note that *θ­*_1_ and *θ­*_2_ are the slopes of each beam element.

The kinetic energy *T* of the structure is given by

(S2)

where *A*_1_ and *A*_2_ are the cross-section areas of each beam element *L_1_* and *L_2_*, *m*_1_ and *m*_2_ are the contact masses considered as lumped masses, and *J*_1_ and *J*_2_ are the rotational inertia of each mass. The position vectors *r_1_* and *r_2_* of each beam motion are given by

$\begin{matrix} r_{1}=\left(u_{1}+x\right)i_{1}+\left(S\left(t\right)+w_{1}\right)j_{1} \\ r_{2}=\left(u_{A}+x_{A}\right)i_{1}+\left(S\left(t\right)+w_{A}\right)j_{1}+\left(u_{2}+y\right)i_{2}+w_{2}j_{2} \end{matrix}$ (S3)

where *i_1_* and *j_1_* are the reference unit vectors of XOY_1_, *i_2_* and *j_2_* are the reference unit vectors of XOY_2_, *S*(*t*) is the base excitation due to the shock, and the subscript *A* is the intersection between the beam elements with respect to *L_1_*, read as

$\begin{matrix} u_{A}=u_{1}\left(x,t\right)|_{x=L_{1}} \\ w_{A}=w_{1}\left(x,t\right)|_{x=L_{1}} \\ x_{A}=L_{1} \end{matrix}$ (S4)

Due to the reference XOY rotating with the cross-section bend angle of the beam, the axis *I* and *j* can be rotated according to

$\left\{\begin{matrix} i_{2} \\ j_{2} \end{matrix}\right\}=\left[\begin{matrix} -\sin\theta_{A} & \cos\theta_{A} \\ -\cos\theta_{A} & -\sin\theta_{A} \end{matrix}\right]\left\{\begin{matrix} i_{1} \\ j_{1} \end{matrix}\right\}$ (S5)

where $\sin\theta_{A}=w'_{A}$ and $\cos\theta_{A}=1+u'_{A}$. In addition, due to the inextensibility of the elements, we have

$(1+u'_{i})^{2}+w'_{i}^{2}=1$ (S6)

where $\sin\theta_{i}=w'_{i}$ and $\cos\theta_{i}=1+u'_{i}$. Since the deflections of the cantilever beam in each section are considered small, we have the following geometrical relationships

$\begin{matrix} \cos\theta_{i}=1+u'_{i}=\sqrt{1-w'_{i}^{2}}=1+O\left(\varepsilon^{2}\right) \\ \theta_{i}=\arcsin w'_{i}=w'_{i}+O\left(\varepsilon^{3}\right) \end{matrix}$ (S7)

Introducing Eq. (S5) into Eq. (S3), taking the derivative with respect to time and multiplication and neglecting the axial displacements, we obtain

(S8)

where we neglected terms of cubic order.

The total potential energy of the structure is given by

$U=\frac{1}{2}\sum_{i=1}^{2} EI_{i}\int_{0}^{l} w''_{i}^{2}ds$ (S9)

Applying Hamilton’s principle with Eqs. (S2) and (S9), neglecting damping, terms above cubic order, and external forces, the equations of motion of the cantilever beam in the strong form are given by

(S10)

where the boundary conditions are obtained as

(S11)

where subscript *B* is the intersection between the beam elements with respect to *L_2_*. Introducing a change of variable for $W_{2}=w_{2}+yw'_{A}+w_{A}$, damping forces, and the electrostatic force applied to the beam element *L_1_* as a partial electrode, the equations of motion become

(S12)

where *c_1_* and *c_2_* are the damping coefficients of each beam element, *l_E_* is the actuation length of the electrode, ε is the dielectric constant, ω is the excitation frequency, V_DC_ is the electrostatic DC load, V_AC_ is the electrostatic AC excitation, and *U* is a unit-step function.

In addition, substituting *W_2_* into the BCs of Eq. (S11) and assuming from the linear free vibration of the 2^nd^ equation of Eq. (S12) that , the boundary conditions can be rewritten as

(S13)

Carrying out a non-dimensional procedure with the new non-dimensional variables as below

$\hat{w}_{1}=\frac{w_{1}}{d}\text{; }\hat{w}_{2}=\frac{W_{2}}{d}\text{; }c_{1}=\frac{2\zeta_{1}\rho A_{1}}{T}\text{; }c_{2}=\frac{2\zeta_{2}\rho A_{2}}{T}\text{; }\hat{x}=\frac{x}{L_{1}}\text{; }\hat{y}=\frac{y}{L_{2}}\text{; }\hat{t}=\frac{t}{T}\text{; }T=\sqrt{\frac{\rho A_{1}L_{1}^{4}}{EI_{1}}};$ (S14)

the equations of motion finally become

(S15)

where Eq. (S15) is the main equations of motion given in the Main text as Eq. (1), and the boundary conditions as

(S16)

where

$\omega_{2}=\sqrt{\frac{h_{2}^{2}L_{1}^{4}}{h_{1}^{2}L_{2}^{4}}};\text{ }\beta=\frac{6\varepsilon L_{1}^{4}}{Eh_{1}^{3}d^{3}};\text{ }\alpha=\frac{T^{2}}{d};\text{ }\Omega=\omega T;$ (S17a)

and for simplicity of calculations, the mass moments of inertia are calculated assuming the switches as rectangular blocks rotating at the beam edge, expressed as

$J_{i}=\frac{m_{i}}{12}\left(a_{1}^{2}+a_{2}^{2}\right)+m_{i}\left(\frac{a_{1}}{2}\right)^{2}$ (S17b)

where *i* is the index for each mass calculated as

$m_{i}=a_{1}\left(a_{2}+\frac{1}{2}\sqrt{a_{3}-\frac{a_{1}^{2}}{4}}\right)$ (S17c)

**S2. Reduced-Order Model and Mode Shapes**

The shape functions ­ related to the cantilever beam of *n* elements and *m* modes of vibration are obtained by solving Eq. (S15) admitting , which yields

(S18)

where $\gamma=\frac{L_{2}}{L_{1}}\sqrt{\frac{h_{1}}{h_{2}}}$, *A­_1,2_-D_1,2_* are unknown constants, *ω_non_* is the non-dimensional frequency, and $\hat{x}$ and $\hat{y}$ are the non-dimensional length of each beam element.

Using Eq. (S18) along with the boundary conditions in Eq. (S16), and accounting for the five modes of vibration for accuracy of the results, the mode shapes of each beam element are written as

(S19a)

(S19b)

(S19c)

(S19d)

(S19e)

and

(S19f)

(S19g)

(S19h)

(S19i)

(S19j)

and are shown in Fig. S2.


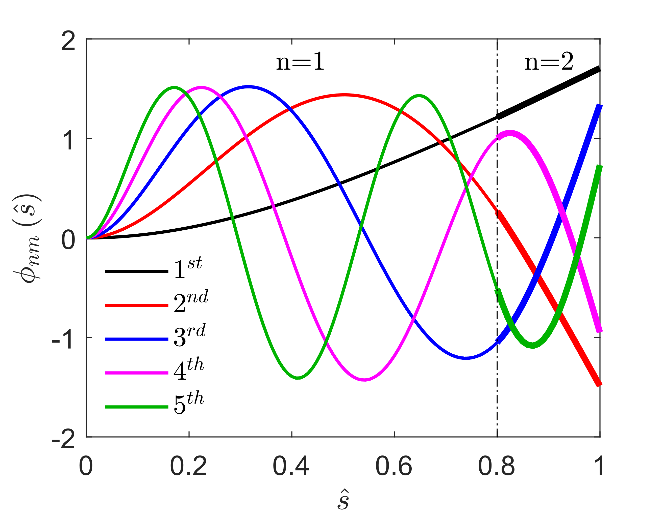


**Fig. S2.** Five mode shape curves at scale of the (a) beam element 1 (n = 1, Eqs. (S19a)–(S19e)), and the (b) beam element 2 (n = 2, Eqs. (S19f)–(S19j)). Each mode of vibration is represented by different colors. Note also that each beam element is presented at a different line width, where the vertical dashed line depicts where both beam elements are linked.

**S3. Measurement of dimensions**

Three samples’ dimensions of the presented MEMS inertial switches were measured using the optical profiler Zygo, and all have yielded very similar results. Fig. S3 shows that the measured thickness of the cantilever beam is 3.3µm. We initially measured the first resonance frequency as 12.375kHz at zero DC voltage.


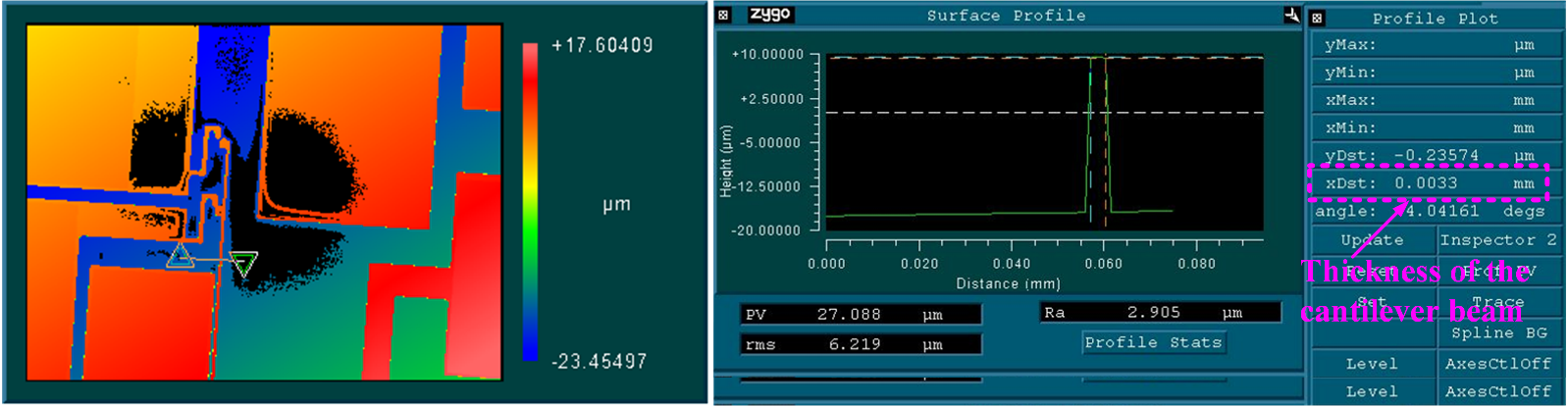


**Fig. S3.** Sample of dimension measurement taken using the optical profiler Zygo.

**S4. Mathematical Model Validation and Quality Factor**

Here, the evaluation of the mathematical model is carried out to determine the quality factor of the microbeam under air environment for the numerical simulations with the shock application in Sec. 5, which is calculated by

$Q=\frac{1}{2\zeta}$ (S20)

where $\zeta=\zeta_{1}=\zeta_{2}$ from Eqs. (S15).

**Fig. S4** shows frequency response curves obtained through the numerical simulations of Eq. (S15) and the experimental data with V_DC_ = 16V and V_AC_ = 1.4V. Excellent agreement is observed. The damping coefficient is then determined as $\zeta=0.0321$, which yields a quality factor *Q* = 15.58. In addition, it is noted that the resonance frequency slightly shifts due to the DC load, which is also matched by the mathematical model.


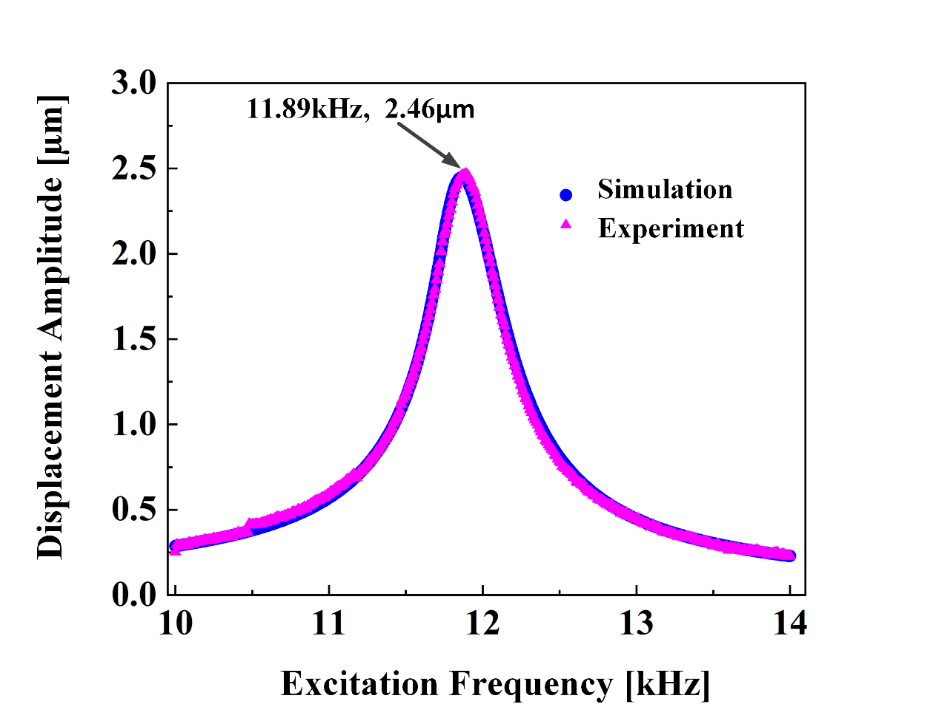


**Fig. S4.** Frequency response curves with V_DC_ =16V and V_AC_ = 1.4V sweeping the frequency around the 1^st^ resonance frequency. The blue circle curve is obtained through numerical simulations of Eq. (S15) while the pink triangle one is obtained throughout the experiments.

**S5. Shock dynamic response discussion**

Fig. S5 shows the ratio of the dynamic response amplification *x_dyn_* over the equivalent static response amplification *x_sta_* as a function of the ratio between the shock force duration and the natural duration of the cantilever microbeam *t_0_/t_n_*. In the regime from A to B, the response experiences large amplification, which is higher than the static response. This range corresponds to the dynamical loading. In addition, the response reaches the peak (1.76) at *t_0_/t_n_*=0.8. When the shock duration *t_0_* is larger than about 5 times the natural duration of the cantilever microbeam *t_n_*, we note that the response of the system is similar for both the dynamic and static loading. It represents the so-called quasi-static regime. In regime beyond B, it is noted that *x_dyn_ / x_sta_* is close to unity as the ratio *t_0_/t_n_* increases. Generally, the natural frequencies of cantilever beams are high due to the small mass. Therefore, their natural periods are very low. They can be easily designed to the large ratio *t_0_/t_n_* which belongs to the quasi-static regime. In this work, the microstructure’s natural period time (0.079ms) is far smaller than the shock duration from the drop-table system, which typically varies within the range of 0.5ms-2ms. Therefore *t_0_/t_n_* ranges from 5 to 50, which corresponds to the quasi-static loading case.


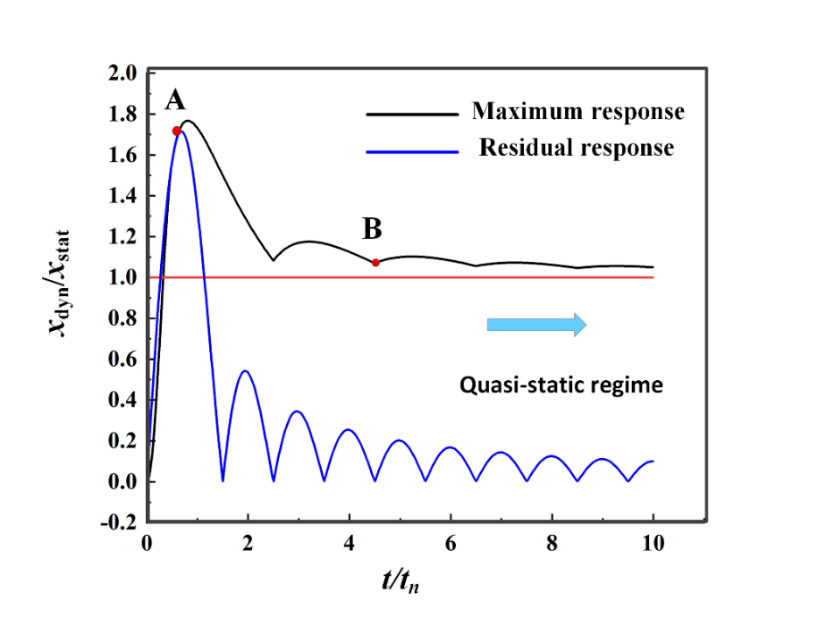


**Fig. S5.** Theoretically calculated results showing a shock response spectrum for the spring-mass system under a half-sine shape acceleration signal.

**Supplementary References**

1. M. I. Younis, “MEMS linear and nonlinear statics and dynamics”, 20, Springer Science & Business Media, 2011.
2. R. T. Rocha, & M. I. Younis, “Nonlinear mode saturation in a U-shaped micro-resonator”, Scientific Reports, 12(1) (2022), 10420.
